# Challenges in Bioinformatics Workflows for Processing Microbiome Omics Data at Scale

**DOI:** 10.3389/fbinf.2021.826370

**Published:** 2022-01-17

**Authors:** Bin Hu, Shane Canon, Emiley A. Eloe-Fadrosh, Anubhav, Michal Babinski, Yuri Corilo, Karen Davenport, William D. Duncan, Kjiersten Fagnan, Mark Flynn, Brian Foster, David Hays, Marcel Huntemann, Elais K. Player Jackson, Julia Kelliher, Po-E. Li, Chien-Chi Lo, Douglas Mans, Lee Ann McCue, Nigel Mouncey, Christopher J. Mungall, Paul D. Piehowski, Samuel O. Purvine, Montana Smith, Neha Jacob Varghese, Donald Winston, Yan Xu, Patrick S. G. Chain

**Affiliations:** ^1^ Bioscience Division, Los Alamos National Laboratory, Los Alamos, NM, United States; ^2^ Lawrence Berkeley National Laboratory, Berkeley, CA, United States; ^3^ Environmental Molecular Sciences Division, Pacific Northwest National Laboratory, Richland, WA, United States; ^4^ Polyneme LLC, New York, NY, United States

**Keywords:** microbiome, microbial ecology, omics, bioinformatics, infrastructure

## Abstract

The nascent field of microbiome science is transitioning from a descriptive approach of cataloging taxa and functions present in an environment to applying multi-omics methods to investigate microbiome dynamics and function. A large number of new tools and algorithms have been designed and used for very specific purposes on samples collected by individual investigators or groups. While these developments have been quite instructive, the ability to compare microbiome data generated by many groups of researchers is impeded by the lack of standardized application of bioinformatics methods. Additionally, there are few examples of broad bioinformatics workflows that can process metagenome, metatranscriptome, metaproteome and metabolomic data at scale, and no central hub that allows processing, or provides varied omics data that are findable, accessible, interoperable and reusable (FAIR). Here, we review some of the challenges that exist in analyzing omics data within the microbiome research sphere, and provide context on how the National Microbiome Data Collaborative has adopted a standardized and open access approach to address such challenges.

## 1 Introduction

The microbiome is defined as a characteristic microbial community occupying a reasonably well-defined habitat which has distinct physio-chemical properties. It includes both the composition of the community (e.g., microbiota) and a theatre of activity, which can be measured with various forms of omics data (Berg et al., 2020). Microbiome research has greatly increased our understanding of the composition and distribution of microbial communities and has provided us with much insight into microbiome functioning, and clues into how best to perturb communities as potential solutions to improve our health and the health of our environment (Donohue and Cogdell 2006; Light et al., 2018; Lear et al., 2021).

While our increased knowledge of individual microbiomes has benefited from a growing number of individual microbiome investigations, the ability to compare data across projects is hampered by many challenges, due in part to the disparate nature of analysis methods employed to process omics data. The ongoing flux in software development and application of new methods to analyze these data have evolved from tackling low throughput technologies (e.g., microscopy) to increasingly high-throughput data, such as metagenomics (Tringe and Rubin 2005), metatranscriptomics (Carvalhais et al., 2012), metabolomics (Bundy et al., 2008), and metaproteomics (Lagier et al., 2018).

Several large-scale microbiome efforts have focused on generating reference genomic data and other valuable omics data (Human Microbiome Project Consortium 2012; Gilbert et al., 2014; Li et al., 2015; Proctor et al., 2019; Parks et al., 2020), yet the velocity at which microbiome data are generated has outpaced infrastructure resources for collection, processing, and distribution of these data in an effective, uniform, and reproducible manner. Given the magnitude of this challenge, there are limited efforts aimed at closing the analysis gap for metagenomic and community profiling data across diverse environments (Gonzalez et al., 2018; Mitchell et al., 2020; Chen et al., 2021). One such effort developed by the European Bioinformatics Institute, called MGnify, provides standardized taxonomic classification of small subunit ribosomal ribonucleic acid gene amplicon data, while for shotgun metagenomic and metatranscriptomics data, MGnify provides assembly, annotation, and contig binning. Importantly, programmatic access to the data for cross-database complex queries is also available via a RESTful application programming interface (API) (Mitchell et al., 2020), and a free service is available for users to submit raw metagenomics sequence data and associated metadata to the European Nucleotide Archive (ENA) followed by analysis using MGnify pipelines. While this platform does not yet support metabolomics and proteomics data analysis, it provides an intuitive way to enable cross-project sequence-based comparisons.

Comparisons across different microbiome studies are of great interest and would allow us to investigate cross-study patterns in a systematic manner to potentially enable generalizable principles to be uncovered. Further, most microbiome studies are underpowered (Kelly et al., 2015), and thus by combining data from different studies, one may find correlations or other associations that cannot be revealed by individual studies alone. For example, it may enable us to differentiate or find similarities in response to various environmental stressors among different microbiomes in different systems. However, several limitations, most notably the broad spectrum (or lack) of metadata standards that allow researchers to find the data they wish to compare, the heterogeneous nature of omics data generated from different labs, and the various data processing/bioinformatics methods, impede the further utilization of these data beyond the scope for which they were originally intended. For researchers interested in cross-study comparisons, it is thus a herculean effort to identify the relevant microbiome studies, to access both the raw omics data and analyzed results, and to re-analyze them in a standardized fashion with other datasets.

To minimize the effort required to identify reusable microbiome datasets, the National Microbiome Data Collaborative (NMDC) was established in 2019 to support microbiome data exploration and discovery through a collaborative, integrative data science ecosystem (Wood-Charlson et al., 2020; Eloe-Fadrosh et al., 2021; Vangay et al., 2021). The NMDC aims to both provide an interface that allows users to search for microbiome samples and omics data based on sample metadata and omics data results, and also provide exemplary open-source analytic workflows for processing petabyte level (10^15^ bytes) raw multi-omics data in microbiome research and producing FAIR compliant (Wilkinson et al., 2016) interoperable and reusable annotated data products. Compared to a typical microbiome study at gigabyte (10^9^) scale, the scope of planned data processing in NMDC represents a 10^6^ fold increase.

Bioinformatics workflows have their own set of requirements compared to the more general and increasingly popular data science practices. For example, the coexistence of different file format standards, various upstream sample collection and preparation methods, and often incomplete sample metadata all require workflow developers to have a comprehensive understanding of both the biology underpinning the analyses, as well as the related statistical and computational methods.

In this paper, we provide a perspective and review some challenges faced since the inception of the NMDC and the implementation of solutions to support standardization and cross-study, cross-sample microbiome comparisons. We believe these challenges and the proposed solutions are applicable to any large-scale bioinformatics or scientific data portal development. We focus on challenges in 1) architecture considerations; 2) microbiome workflow selections; 3) Metadata to standardize and manage workflow data products.

## 2 Architecture Considerations

**FIGURE 1:**
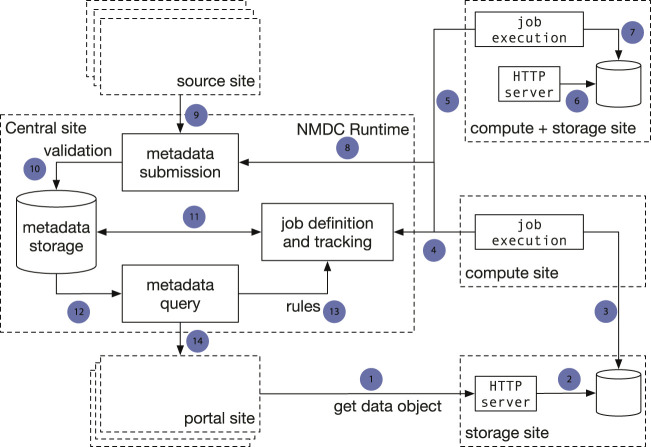
Implementation of a data federation model in the NMDC pilot. The central site implements the NMDC Runtime API that orchestrates the data flow with a database that serves as the data registry. The Runtime validates submitted metadata against the NMDC schema and detects new jobs to be done based on submitted-data annotations. Source sites submit raw experimental data and sample metadata to the central site. Compute sites poll the Runtime for new workflow jobs to be done, claim jobs appropriate for their capabilities, and submit workflow job outputs to the central site. Storage sites store raw workflow outputs. The portal site provides a web-based interface. One site can serve as both a computing site and storage site. Arrows: 1): Portal site gets data object from HTTP server at a storage site; 2): The HTTP server retrieves data from a database; 3) A compute site deposits workflow run result data to a database at a separate storage site; 4) Compute sites claim computing jobs and provide job execution updates to the job tracking mechanism at the Central site; 5, 6, 7): A compute site can also serve as a storage site at the same time; 8) Compute jobs are associated with the sample metadata; 9) A source site submits sample metadata to the Central site; 10) Central site validates submitted sample metadata; 11) New jobs are created from the submitted samples metadata and become claimable by compute sites; 12) Sample metadata can be queried; 13) A set of rules define the type of computing jobs that can be claimed by every Compute site; 14) The Portal site queries metadata.

There are two major architecture patterns for data portal design, namely data warehouse (Gardner 1998; Koh and Brusic 2005) and data federation (Haas et al., 2002). Though both patterns support multiple sources to submit data, the major difference is that with data commons all the data storage, analysis, and access are provided through a single location instead of from different participating sites. To avoid duplicating data from its submitters, the NMDC adopts the data federation pattern. The NMDC participating institutions can serve as satellite sites, which can be further categorized by its function as experimental site (where raw experimental data are generated), computing site (where bioinformatics workflows are executed), storage site (where raw and/or workflow output data are stored) or any combination. There is a separate central site that functions as the central registry to maintain a global catalog of metadata and data and to link a set of heterogeneous data sources. The central site implements an application programming interface (API) that allows search of the data and communication with satellite sites. It also hosts the web portal (Figure 1). A new institution can join the NMDC data federation by registering as a satellite site and implementing protocols that communicate with the NMDC API. Adopting the data federation pattern allows different sites to maintain their own computing environment setup indepently, e.g., using different job management solutions, such as SLURM (Yoo et al., 2003) or Univa Grid Engine (https://www.altair.com/grid-engine/), which also brought us some additional considerations for workflow designs. It also provides the flexibility to bring bioinformatics workflows (gigabytes in size) to experimental and storage sites, instead of moving raw omics data (often terabytes or even larger in size) to a compute site. This model also allows experimental data generation sites to integrate with local data services used for tracking critical metadata and automatically submitting data into the central registry. The current NMDC sites are tightly coupled through the development of the NMDC as the original infrastructure developers, however future NMDC satellite sites can be more loosely coupled as they will not be responsible for maintaining the core infrastructure. Instead, these satellite sites will maintain data processing and exchange services based on their needs to connect with the NMDC project.

## 3 Microbiome Omics Workflow Considerations

As an increasingly varied array of omics data are being generated for more and more microbiomes, the NMDC team supports standardized workflows for the consistent analysis of metagenomics, metatranscriptomics, metaproteomics, and a suite of metabolomics data. Open-source bioinformatics workflows for processing raw multi-omics data have been developed based on production-quality workflows at the two Department of Energy User Facilities, the Joint Genome Institute (JGI) at Lawrence Berkeley National Laboratory (LBNL) and the Environmental Molecular Sciences Laboratory (EMSL) at Pacific Northwest National Laboratory (PNNL). For any given set of omics data processing or analysis, there exist many tools that typically undergo frequent updates as technologies advance. To accommodate the goals of providing an expanded search capability for NMDC users, the primary goal was to deliver a scalable, open source platform that could provide standardized results independent of the computing platform used, thereby accelerating and enabling future downstream comparative microbiome analytics. It is also worth noting that to help standardize the workflow outputs for cross-study comparisons, we have specified the parameters used in all the NMDC workflows. In other words, all the workflows are static to keep output consistency. Given the various experimental instruments for generating any of these omics data and the associated complexities of instrument-specific biases and error models, we decided to initially focus on the most popular methods applied to microbiome samples developed and maintained by the JGI and EMSL, including Illumina sequencing data, bottom-up proteomics using data-dependent acquisition (Stahl et al., 1996; Kalli et al., 2013), gas chromatography mass spectrometry (GC-MS) based untargeted metabolomics (Hiller et al., 2009; Fiehn 2016) and Fourier transform ion cyclotron resonance mass spectrometry of complex mixtures (FT-ICR MS) (Kujawinski 2002; Ghaste et al., 2016; Corilo et al., 2021) data, in our initial workflow implementations and software package releases. A Liquid chromatography–mass spectrometry (LC-MS) based workflow is under development and will be available later this year.

### 3.1 Common Assumptions in Workflows

Many of the challenges in bioinformatics workflows relate to various assumptions made by the workflow software developers. Typically, a bioinformatics workflow tool is developed to solve a data analysis need for a specific experimental design, as well as specific data types and volumes generated for a specific project and to be run within a specific computational infrastructure. Adaptation of specific bioinformatics tools or workflows for a broader project such as that embarked upon by the NMDC requires a more thorough analysis of the workflow requirements and portability needs. The result is a solution that cannot readily be separated from the developers’ computing environment with various explicit and implicit assumptions, such as the availability of specific job scheduler and compiler, Linux kernel module and even a specific distribution, instrumentation, file naming conversions, and storage location and formats of the input and output files. We have also investigated the memory usage requirements of various software components, particularly metagenome assemblers, which are known for their high-memory requirements (Kleftogiannis et al., 2013; Li et al., 2015). Another implicit but common assumption is that workflows are for scientists or humans to execute manually on a handful of datasets, instead of being automated for many thousands of datasets, and actively monitored by software, which is linked to workflow scaling.

### 3.2 Scaling Workflows

Scaling in bioinformatics workflows means the process of dynamically adjusting compute, storage, and network services to meet the data processing demands in an automated fashion in order to maintain availability and performance as utilization increases. Scaling is a common design requirement in cloud applications and has begun to attract attention from the bioinformatics community. Scaling is usually not a requirement for workflows designed to serve small to medium scale studies (with perhaps a few terabytes of raw data) since these workflows can be started manually and queued in a shared job environment. However, in large-scale studies, workflows are being used as a service and must be automatically triggered based on the detection of the availability of new experimental data and additional computing resources may need to be added without interruption to existing workflow executions (Clum et al., 2021). Also, large-scale studies often involve several experimental laboratories and data may be processed at different computing sites, which may run different job schedulers. Thus, it is important that job schedulers have to be separated from the workflow implementation and be configurable for each computing site. For example, based on the raw sequencing data size and complexity, the *de novo* assembly of metagenomic and metatranscriptomic data often requires access to high memory (>1 terabyte) computing nodes. An algorithm is needed to estimate the memory and time needed to process a given sequencing dataset and only allow a data processing site with available big memory nodes to claim such jobs. When cloud resources are used, the appropriate virtual machine instance with sufficient memory and storage must be instantiated. Within the NMDC, a runtime API (https://microbiomedata.github.io/nmdc-runtime/) was implemented that constantly monitors the raw data availability, raw data type (which decides which workflow needs to run), and the computing resources available at each computing site (Figure 1). The runtime API is based on the Global Alliance for Genomes and Health GA4GH Data Repository Service (DRS) standard (Rehm et al., 2021). Some other scaling related issues are listed in Table 1.

**TABLE 1:** Scaling related considerations.

	Small scale studies (gigabytes to <10 terabytes)	Large scale studies and data portals (>10 terabytes)
Workflow management	Rarely used, typically job scheduler	Dedicated workflow manager program
Workflow reproducibility	Limited reproducibility within developers’ specific computing environment, lack of long term support	Reproducible independent of the computing environment, better support
Metadata management	Usually at intra-study level and nonstandardized	Community standard based and enforced
Data Management	Spreadsheets	Databases with API access
Data Query	Manual lookup	Database queries and APIs

### 3.3 Selection of Workflows Based on Best Practices

Based on NMDC expertise and general knowledge of the bioinformatics landscape for varied omics data analysis software, no available workflows could accommodate our design needs, e.g., scalability, portability, and reproducibility. While there is no ultimate gold standard workflow for performing environmental microbiome omics analyses, the metagenomics, metatranscriptomics workflows developed at the JGI and the metabolomics and metaproteomics workflow developed at EMSL have been rigorously tested with hundreds and thousands of datasets in the past decade. These workflows, though developed with the assumptions about their local computing environments and not easily portable, do cover a variety of memory and parallelization requirements and follow some of the best practices, and were chosen as the foundation of the NDMC workflows (Piehowski et al., 2013; Li et al., 2017; Clum et al., 2021; Wu et al., 2021). We have introduced several enhancements on top of these foundations. Firstly, to make these workflows fully portable and scalable, we have removed or abstracted all computing environment dependencies by containerizing all the software components. Secondly, we implemented all the workflow logic using the workflow definition language (WDL) (Voss et al., 2017). We also added standardized workflow output file formats in a schema to verify workflow outputs are ready for data ingestion, described further below in the Workflow Metadata section. To help external users adopt these workflows and run them within their own computational environments, we have put all the workflow definitions with test datasets in the NMDC project Github organization (https://github.com/microbiomedata). In addition to this open access software, we further provide detailed documentation (https://nmdc-workflow-documentation.readthedocs.io/en/latest/). Additional training materials are also provided, including video instructions on using the NMDC portal site to examine processed data, and how to run the NMDC workflows in the NMDC EDGE web application (https://nmdc-edge.org), which provides access to all available NMDC omics workflows and is open for public use.

### 3.4 Workflow Manager and Workflow Definition

Containerizing workflow components and adopting a workflow definition language alone are not sufficient to separate the concerns of workflow logic and its execution environment. A workflow manager is still required to cleanly separate the concerns of workflow definition and workflow execution. Compared to traditional pipelines utilizing job schedulers or scripting languages, workflow managers excel at reproducibility, data provenance and portability (Di Tommaso et al., 2017; Wratten et al., 2021). Each data processing site only needs to install and configure its own data workflow manager instance based on its resources, such as memory, CPUs, job queues, and storage. With detailed information retrievable from the workflow manager’s database, information about workflow execution status is no longer limited to the computing system’s job queue itself (e.g., Slurm). This also provides support for resuming failed workflow executions from where the workflow stopped instead of at the beginning of the entire workflow.

**FIGURE 2:**
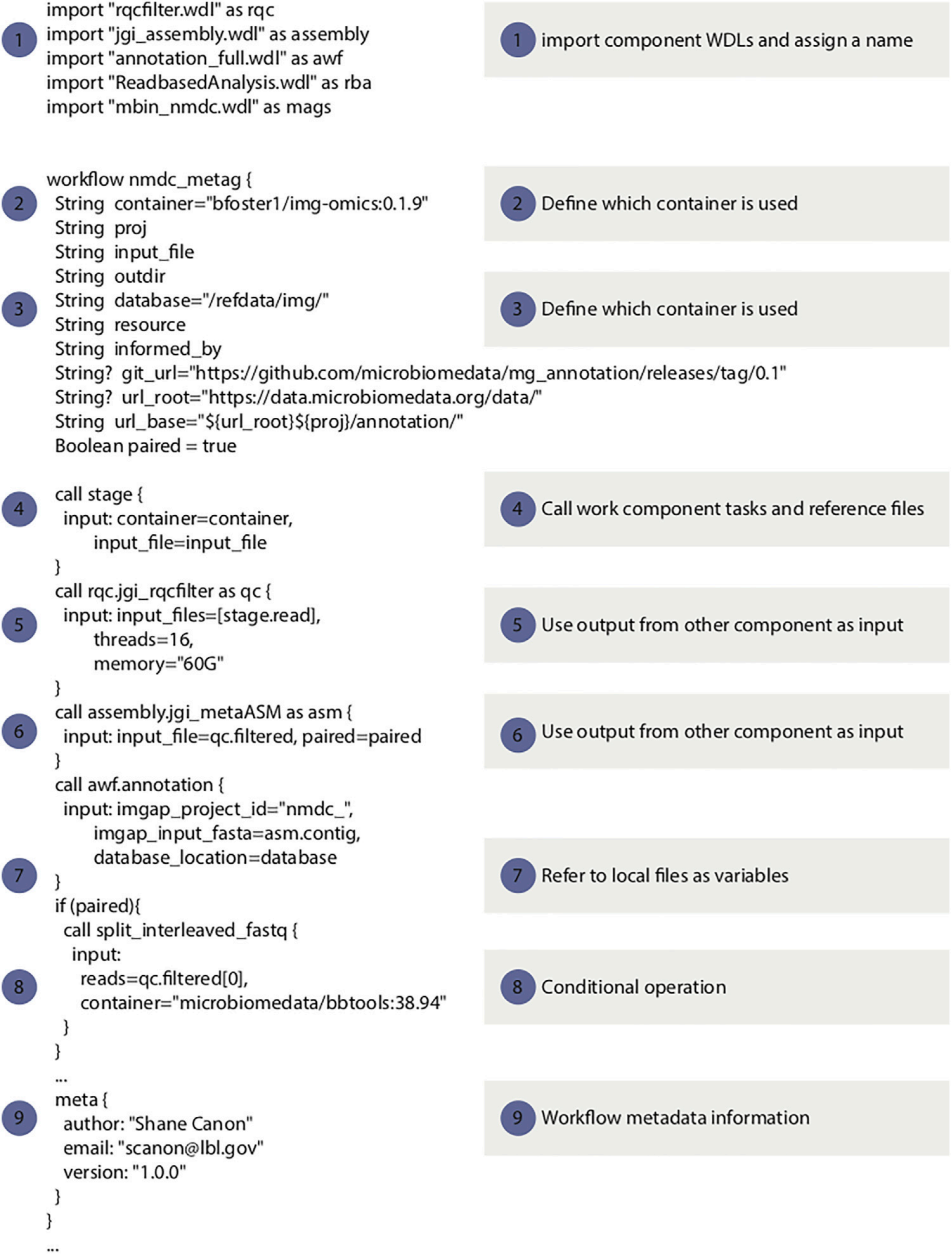
Code snippets of the metagenomic data workflow to illustrate the WDL best practices listed in this paper 1: example use of the “import” function (best practice point 1); 2–4: examples of using containers in WDL (best practice points 2-4 and 6); 5–8: examples of avoid site specific implementation (best practice point 4); 9: workflow metadata information (best practice point 8). The full workflow code is available from https://github.com/microbiomedata/metaG.

For the NMDC, WDL was selected over other workflow languages primarily based on reusable workflow components and superior standardization, which has also been reported by others (Perkel 2019). The Cromwell workflow manager is used in the NMDC due to its native support for WDL (Voss et al., 2017). Cromwell also provides a rich set of features including existing support for a variety of batch systems, native support for containers, “call-caching” to reuse previously executed tasks and an API to facilitate automation. Several key best practices that were adopted by the NMDC for specifying workflows using WDL with component software packaged in containers are listed below. Figure 2 displays a snippet of WDL code from the NMDC metagenomics workflow, to highlight several key considerations when developing WDL code.

(1) Utilize the WDL “import” function to break down the complexity in large workflows to smaller components. This makes the workflow maintenance easier and increases components reuse.
(2) All workflow tasks should use containers to improve portability, consistency and reproducibility.
(3) All container images should have published recipes (e.g. Dockerfiles). This makes it easier for others to understand how an image was generated and make modifications if needed.
(4) The WDL files should not include any site specific implementation. The Cromwell configuration file should be used to handle site integration. This ensures the WDL is as portable as possible.
(5) Workflows should avoid doing major pre-processing or post-processing outside the WDL. All of the major analysis should be captured in the WDL. This makes the analysis more transparent. For example, generating gene expression information has to be part of the WDL.
(6) Container images should be versioned and the version should be specified in the WDL. This makes the workflow more transparent and ensures that the tasks specified in the WDL are in sync with the image contents. For example, if a new tool version is used that has different command-line options, the WDL and image version can be changed in sync with one another.
(7) Reference data should be versioned and the workflow should specify which version of the reference data is to be used. This avoids potentially format mismatches and helps with reproducibility and transparency.
(8) Workflow WDL have to provide a metadata section that includes the workflow version and author information.

### 3.5 Workflow Deployment

One of the common challenges in complex bioinformatics workflows is how to best resolve the conflicting software dependencies, and managing the versioning of component software. For NMDC, we addressed this challenge by separating the workflow definition and its runtime by the adoption of workflow managers and WDL as described in the previous section. We also packaged all the runtime requirements for each workflow in Docker containers (Merkel 2014) and made them freely available to non-commercial users (https://hub.docker.com/u/microbiomedata). Some of the runtime components are developed by third parties and have restrictions for commercial users. However, researchers can still use the NMDC workflow WDL definitions by either acquiring appropriate component software licenses (free and open to non-profit organizations and universities). Currently, the NMDC workflows have been deployed to NMDC partner organizations: the National Energy Research Scientific Computing Center (NERSC), the Environmental Molecular Sciences Laboratory (EMSL), the San Diego Super Computing Center (SDSC), and the Los Alamos National Laboratory (LANL). Adding another data processing site or running it in a local computing environment only requires installing and configuring Cromwell and Docker. In high performance computing (HPC) environments where elevated user privilege is a concern, the NMDC workflow containers have also been adapted to other software container solutions that are HPC-friendly, e.g., Singularity (Kurtzer et al., 2017), Shifter (Jacobsen and Canon 2015), and Charliecloud (Priedhorsky and Randles 2017).

Through our experience in packaging and testing of these workflows in different environments, we have learned a few lessons. One is user privilege management. For example, the Cromwell user account needs to have access to all the virtual storage volumes used by the workflow runtime containers. Also, when packaging tools into WDL, workflow developers should really avoid writing to the “/tmp” directory in containers since default settings of writing to “/tmp” is prohibited in Singularity and Charliecloud containers, while being allowed in Docker and Shifter. For testing purposes, we also suggest a minimum of including two testing data sets for each workflow. One smaller test data set can be used for rapid workflow logic validation. The second data set should be complex enough to test memory usage and allows for benchmarking and estimation of CPU and memory usages. In addition, when building software in the containers, chip specific instructions have to be avoided in order to maintain portability. This can mean trading off performance for portability. We have evaluated our workflow containers in both physical and virtual environments running on Intel and AMD processors. We have tested these workflows in various HPC facilities (NERSC/DOE, Expanse/San Diego Supercomputing Center, Texas Advanced Computing Center, Los Alamos National Laboratory, Environmental Molecular Sciences Laboratory/DOE). For some workflows that do not require large memories or databases to run, we have also tested on laptop computers. The NMDC continues to evaluate support for non-x86-64 architectures based on support of the underlying tools and the prevalence of these systems within the community. Presently, many of the underlying tools have not been tested or optimized for architectures such as ARM64 or PPC64 so supporting these is not in any near-term plans. Likewise, GPU support in most of the tools is limited or non-existent. We will continue to track any improvements and make adjustments in the NMDC workflow and images as these tools and community access evolves.

### 3.6 Workflow User Interface and Customization

The intended users include all microbiome researchers, including both bench scientists and bioinformaticians. To assist bench scientists to use the NMDC workflow, we have carefully engineered a web-based user interface (NMDC EDGE) to run the NMDC workflows interactively (https://nmdc-edge.org). Since we aim to provide a catalogue of the existing microbiome data based on unified analysis processes, we made a design decision to use static workflows with fixed parameters for all the data that feeds into the NMDC portal. Customized workflow runs, including changing the default workflow parameters and even modified the workflow components for internal analysis needs that are not submitted to the NMDC portal will be supported through the KBase (https://www.kbase.us) and future versions of the NMDC EDGE.

## 4 Metadata

Metadata in the NMDC includes both sample metadata that describes the origin and environmental attributes of the biological sample collection, as well as metadata related to the omics analysis processes employed. The NMDC schema controls which metadata elements are applicable or required for all data within the NMDC, whether it is sample data, or data generated from workflows. The NMDC schema is defined using Linked Data Modeling Language (LinkML, https://linkml.io/linkml/). LinkML is a rich modeling language that is used to create schemas that define the structure of data, allows for rich semantic description of data elements, as well as leveraging JSON-Schema for validation. For example, the relationship between studies, samples, workflows, and data objects is described using LinkML, and the metadata dictionary for samples is described using LinkML.

For sample metadata, our schema leverages the Minimal Information about any Sequence (MIxS) data dictionary provided by the (Genomics Standards Consortium (GSC, https://gensc.org/mixs/) (Yilmaz et al., 2011), as well as environmental descriptors taken from the Genomes Online Database (GOLD) (Mukherjee et al., 2021), and OBO Foundry’s Environmental Ontology (EnvO) (Buttigieg et al., 2013).

**FIGURE 3:**
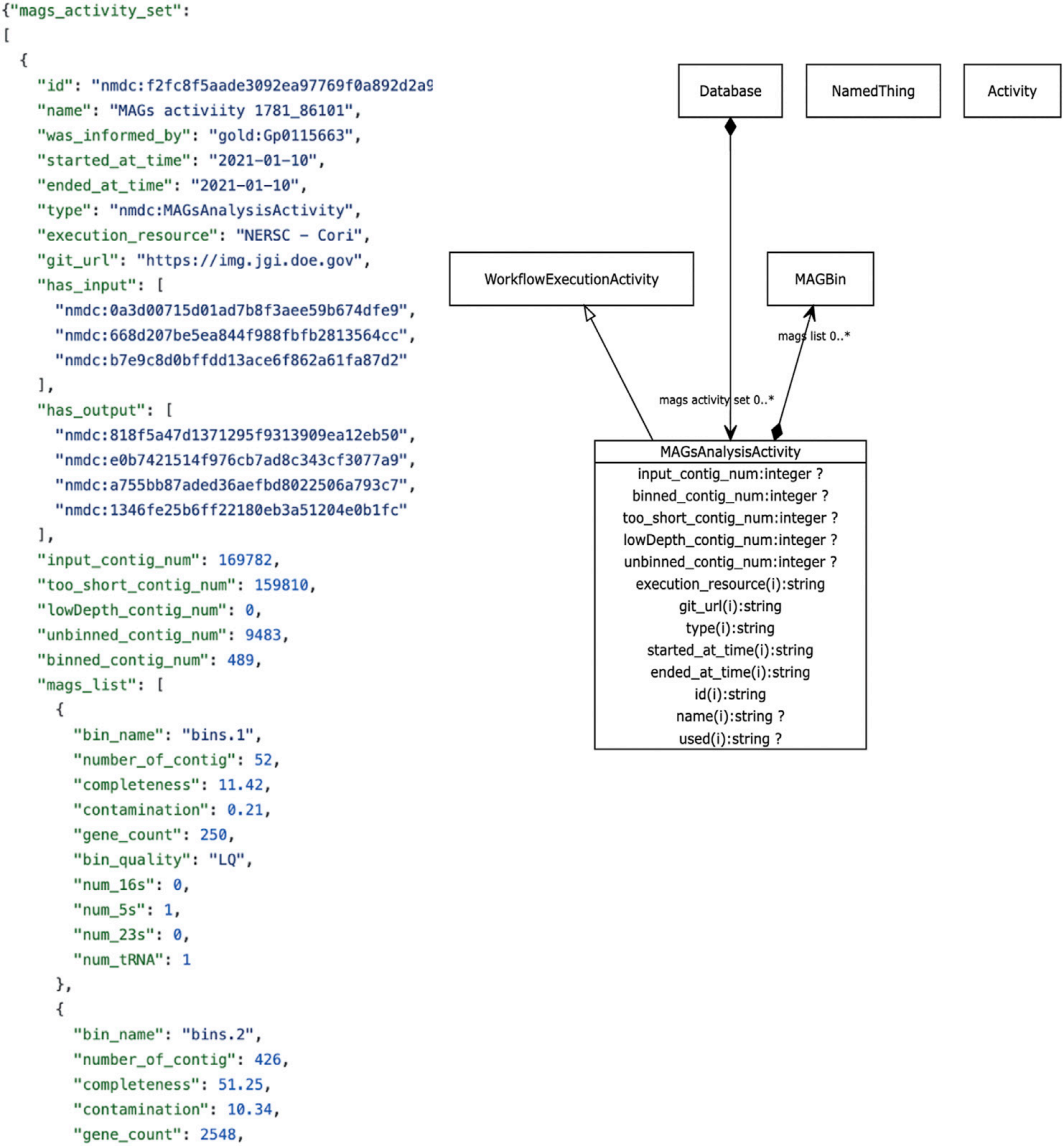
Example NMDC workflow metadata. Left panel shows an example JSON output snippet of a MAGSAnalysisActivity, which is a record of the metagenomic assembled genome (MAG) workflow execution. It includes generic workflow metadata (start/end time, execution resource) and MAG-specific metadata and workflow outputs. The full JSON example is available on-line (https://github.com/microbiomedata/nmdc-metadata/blob/master/examples/MAGs_activity.json). Right panel shows a visual depiction of the MAGSAnalysisActivity class in the NMDC LinkML schema (https://microbiomedata.github.io/nmdc-schema/MAGsAnalysisActivity/).

The metadata we focus on in this review revolves around descriptive metadata on the procedures used to generate and process the data. The NMDC leverages the PROV ontology standard (https://www.w3.org/TR/prov-o/), which is a well-established practice in the semantic web community, to provide provenance information. Instances of workflow runs are represented as PROV *activities*. We include distinct schema classes for workflow executions such as Metagenome Assembly, Metabolomics Analysis Activity, Metagenome Annotation Activity, etc. Each of these has generic metadata associated such as time of execution, site of execution, inputs, outputs, etc., in addition to metadata specific to each type of workflow. For example, metabolomics activities have metadata such as calibrations, metabolite quantifications, instruments use. Where possible, these descriptors are mapped to existing standards and vocabularies. An example is provided in Figure 3. A Uniform Resource Identifier (URI) and associated workflow activities have been assigned to all the workflow output files that are ingested to the backend database. In the example for Figure 3, the URI “nmdc:MAGsAnalysisActivity” is assigned for the outputs of the metagenome binning workflow. This approach lays the foundation for checking workflow output integrity and it also helps to guide the user interface development decisions for the portal website (e.g., what types of searches will be allowed).

Metadata can also be used to steer the workflow execution. For example, nucleotide sequencing data generated from the Illumina platform can be either single-ended (SE) or paired-ended (PE) and PE reads can be stored either in two separate fastq files or interleaved in one fastq file, which makes three types of potential input formats for the NMDC metagenomic and metatranscriptomic workflows. The NMDC workflow supports both SE and PE reads. We plan to automate the detection of the SE/PE reads and leverage the sample preparation and instrumentation metadata to set parameters to the workflow and to trigger appropriate workflow component tasks.

## 5 Summary and Future

Here, we have outlined some of the challenges and considerations in implementing disseminable standardized bioinformatics workflows for microbiome omics data, with the goal of providing microbiome analyses that may be cross-compared across projects, regardless of the samples, or the computational environment used to generate the results. The initial efforts from the NMDC have shown how some of these challenges can be addressed by adopting workflow managers, workflow definition language, containerizing workflow runtimes, and developing a data schema for workflow files and their contents. The NMDC has also adopted a data federation model to allow multiple sites (as either data generation, computing, or data storage sites) to contribute to the NMDC while minimizing resource challenges on any single site.

While we document progress towards robust and standardized analyses of various microbiome omics data types, many challenges remain. For example, the data flow in the NMDC is not yet entirely automated which would significantly increase processing capacity. The NMDC is developing a runtime API to fully automate the processing of microbiome data and that supports continuous integration and continuous development. We are also actively developing and supporting a public-facing NMDC API. We have already implemented a set of APIs for satellite sites to register samples and submit workflow outputs in JSON to the portal. Currently, this API is used by the NDMC developers and will be open to external developers. We also plan to provide a set of APIs for programmatic data access, such as query and import data from the NMDC (e.g., the KBase project plans to provide this utility from within the KBase platform). Concomitantly, the optimization of workflow parameters based on sample metadata is also being undertaken, which would then further support automation.

The NMDC data that have been integrated thus far have been generated from JGI and EMSL, both of which serve as experimental data generation sites, compute sites, and storage sites. Separate omics data processing workflows have been developed, and the integration for these data happens through the harmonization of the sample metadata and the functional annotation information. Specifically, the metagenomics, metatranscriptomics, and metaproteomics workflows rely on the same underlying annotations to allow cross-comparisons. At this time, the integration of metabolomics data is only available through the sample metadata information. The workflows and infrastructure envisioned to process future microbiome data, including all microbiome data stored in the short read archive (SRA), are envisioned to be deployed as omics analysis platforms as a service (PaaS) in the cloud for prompt data processing. The NMDC team will coordinate with the user to decide the best strategy to process or deposit a large amount of data.

Lastly, one of the largest and likely ever-present challenges that remains surrounds the topics of sustainability and updating results. These must be considered given the constantly changing landscape of our knowledge of the biological world, and the tools and technology (both instruments and algorithms) used to interrogate microbiomes. Workflow extensibility and database version reliance regularly factor into workflow design considerations. Like almost all bioinformatic workflows, the NMDC workflows rely on several reference databases for genome annotation, metagenome-assembled genome binning, taxonomy classification, and protein and metabolome assignments. As a result, alternative databases, or updates to any of these databases can lead to differences in workflow outputs, which engender important considerations: when to reprocess sample data and how to control the versions of the workflows, databases, and their outputs. An open question is when it is necessary to rerun the analysis on all or a subset of microbiome omics data to update the analysis results. A rerun of the workflow may be triggered by a major update in either the database or the workflow itself. For example, a major change in the NCBI taxonomy database, which is used to identify taxa within metagenomic and metatranscriptomic data, would warrant reprocessing samples affected by these changes. Similarly, newly discovered genomes could enhance both taxonomy and annotation results, or new discoveries in protein structure and function or new metabolites may require reanalysis of metaproteome and metabolomic datasets as well. In addition, new or improvements in algorithms and software tools that significantly outperform existing tools will likely require rerunning relevant workflows.

The NMDC model, in terms of workflow development, implementation, and sharing has been to construct modular workflows from established, best practice tools and pipelines, and to make these open source (https://github.com/microbiomedata). While the NMDC plans to use these workflows to process currently available microbiome data, continued testing and evaluation of new tools, or new versions of existing tools is also underway and part of our internal processes and policies related to workflow management, updates, and adoption of new tools. Specifically, the current NMDC workflows are derived from established workflows from the NMDC participating organizations and will evolve over time. These upstream workflows will routinely undergo modifications in order to improve the quality and performance of the results and products, through the adoption of new tools, updates to the various software packages, updates to the reference databases and taxonomy, etc. The NMDC will synchronize with the upstream workflow changes to improve the NMDC workflows. The open source model also allows third party developers to substitute tools and examine how changes to the workflows may impact the results. As the NMDC team further develops support for curated metadata and production-quality bioinformatic workflows, we welcome contributions from the broader community of researchers to partner with us to make the NMDC a unique collaborative resource for microbiome researchers.
